# FIRST PASSAGE TIMES TO T CELL ACTIVATION

**Published:** 2025-10-02

**Authors:** TONY WONG, IKCHANG CHO, MARIA R. D’ORSOGNA, TOM CHOU

**Affiliations:** * Department of Mathematics, University of California, Los Angeles.; † The University of Chicago.; ‡ Department of Mathematics, California State University at Northridge.; § Department of Computational Medicine, University of California, Los Angeles.

**Keywords:** First-passage times, adaptive immune system, antigen recognition, T cells, kinetic proofreading, 35K57, 35Q92, 60J70, 92C17, 92C37

## Abstract

Effective recognition of foreign antigens by the adaptive immune system relies on T cells being activated by antigen-presenting cells (APCs) in lymph nodes. Here, diffusing T cells may encounter cognate APCs that present matching antigen fragments or non-cognate ones that do not; they are also subject to degradation. We develop a stochastic model in which T cell-APCs interact via a sequence of recognition steps, represented as a multistage Markov chain. T cells are successfully activated only if the terminal state associated with a cognate APC is reached. We compute the probability of successful activation in the presence of interfering non-cognate APCs, T cell degradation, and lymph node exit, and analyze the mean first-passage time to activation. We also incorporate a kinetic proofreading mechanism that enables state resetting, and show how this enhances specificity toward cognate APCs.

**Relevance to Life Sciences:**

We present a quantitative framework to study T cell activation within the lymph node that integrates diffusion, the presence and abundance of cognate and non-cognate APCs, and T cell death and exit from the lymph node. Relevant spatio-temporal parameters, such as T cell diffusivity and residence time within the lymph node, are estimated from existing literature. Quantification of the activation probability and time to first activation provide fundamental insights into the onset of the adaptive immune response.

**Mathematical Content:**

T cell recognition is modeled as a multistage Markov process, coupled with spatial diffusion, exit, and death. All four processes are represented through a system of partial differential equations that are analyzed under Robin and Neumann boundary conditions. Using first-passage time theory, we calculate activation probabilities and mean activation times. We also show how kinetic proofreading through stochastic resetting enhances specificity.

## 1. Introduction and Background.

The adaptive immune system plays a central role in defending an organism from disease. Key components are antigen-presenting cells (APCs) and T cells that co-localize in lymph nodes where they interact to trigger proliferation or B cell signaling [45]. APCs capture antigens, short amino-acid sequences that are part of larger proteins, from foreign agents encountered throughout the body. Enzymatic degradation turns these antigens into smaller peptides that are presented to the major histocompatibility complexes (MHCs) on the APC surfaces for T cells to recognize.

T cells constitute a highly diversified population. Each of them expresses a specific surface receptor (TCR) that can only recognize a small subset of “cognate” antigens. Successful recognition involves several biochemical interactions between TCRs and the antigen-loaded MHCs that include conformational changes which in turn trigger downstream signaling events. Upon recognition, naïve T cells become activated and initiate an immune response by proliferating and differentiating into effector T cells such as cytotoxic T cells that eliminate pathogens, and helper T cells that activate other immune cells; most of them migrate from the lymph node to peripheral tissues to kill the infected host cells. Some activated T cells become long-lived memory cells that help trigger an effective and rapid immune response if the same pathogen is encountered again. Each T cell is associated to a small number of cognate APCs (cAPCs), all the others are not recognized and are referred to as non-cognate APCs (nAPCs). It is estimated that a given APC is cognate to one in 10^5^–10^6^ T cells [5,26,30,57]; the inverse of this quantity is known as the precursor frequency. Given how rare it is for a T cell to encounter its cAPC, the problem is sometimes known as that of “searching for a needle in a haystack” [38].

Most T cells are produced in the bone marrow, mature in the thymus, and are transported via the bloodstream to the approximately 600 lymph nodes in the human body. T cells are in continuous recirculation: from the blood they reach a lymph node and search for their cAPC. If the search is not successful within 12 to 24 hours, they return to the bloodstream, enter a different lymph node and repeat the cycle [18,27,40,53]. The lifespan of naïve, unactivated T cells ranges from weeks to years, depending on age and health status [6,54]. On the contrary, once a foreign antigen has been acquired, an APC will migrate from the exposed tissue (such as the skin) to the closest lymph node through lymphatic vessels and remain there [47]. The typical lifespan of an APC within a lymph node is two to six days [29,51].

Recent advances in 3D imaging, flow cytometry and quantitative PCR have been used to shed light on how T cells interact with APCs, particularly dendritic cells [19, 44]. Most T cells and APCs co-localize in the “T cell zone,” a specialized sub-compartment that occupies a large portion of the lymph node [2]. T cells within this compartment do not follow chemotactic gradients but instead encounter antigen-presenting cells (APCs) through independent, random motion. Their movement is guided by an underlying network of fibroblastic reticular cells, which provides structural support to the lymph node and helps organize cell trafficking within it [1,28,33,38]. The speed, persistence times, and turning angles of T cells have been quantified, revealing that within the T cell zone, T cells are much more motile than dendritic and B cells [39,52]. In addition, dendrites emanating from the cellular core of dendritic cells are highly dynamic, increasing their effective spatial extent. As a result, the contact frequency between T cells and APCs is elevated, leading to an efficient scanning process [56]. For example, a dendritic cell can engage with up to 80 T cells per minute with the typical contact between a T cell and a nAPC lasting roughly 3 minutes before dissociation [37]. Upon encountering a cAPC, however, a T cell will arrest its motion to allow for biochemical and conformational changes that stabilize the low-affinity TCR-MHC contact to take place; this association can last more than 15 hours before the immune response is triggered [50].

Mathematically, the movement of T cells in lymph nodes has been studied via computational models based on two-photon microscopy imaging [3,4,12,15,41]. For example, the residence time of a T cell interacting with a nAPCs has been fitted to an exponential distribution whose mean depends on the specific T cell type [35]. Other theoretical studies propose different types of random walks, such as Brownian motion [7,11], Lévy walks [20], and velocity-jump models [46]. Typically, cAPCs are modeled as a finite set of small, stationary, and well-separated target sites; encounters are defined as a T cell reaching or coming within a given distance from these sites [7]. In these studies, the presence of nAPCs is often neglected [10,31,36]. The probability of a T cell encountering its cAPC within a fixed time has also been used to estimate the likelihood of initiating the adaptive immune response [7,46].

## 2. Mathematical model and analysis.

**Fig. 1:**
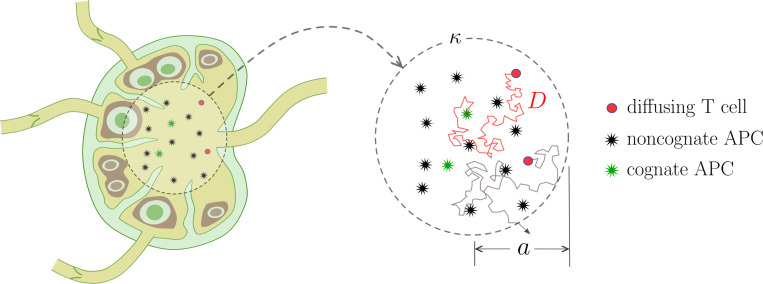
Spatial representation of our model. A schematic of a lymph node is shown on the left. The T cell zone, where T cells (red dots) diffuse and encounter cognate (green stars) or noncognate (black stars) APCs, is approximated as a sphere of radius a in the schematic on the right. The diffusion constant D is assumed to be uniform. Removal of T cells occurs via two mechanisms: exit through the boundary of the T cell zone, and death or egress from the interior. The latter process is facilitated by specialized lymphatic channels not shown.

We develop a mathematical framework to study antigen induced T cell activation where the presence of both cognate and non-cognate APCs are explicitly included. Other features are T cell death and egress from lymphatic channels, the relative scarcity of cAPCs compared to nAPCs, and non-trivial activation mechanisms such as multi-stage binding or kinetic proofreading. We quantify the statistics of the conditional cAPC-induced activation times of T cells [11] in the dominating presence of nAPCs. T cells are assumed to be point particles that diffuse in a three-dimensional, spherical lymph node compartment, uniformly populated by APCs. Quantities of interest include the probability that a T cell is activated by its cAPC before exiting the T cell zone, and the conditional first passage time to full activation [9,14,25,48]. The overall geometry of our model is shown in Fig. 1, where for simplicity the T cell zone is modeled as a sphere.

In section 3 we present a reversible, multi-stage, two-arm model, in which a T cell diffusing within a sphere can bind to either a non-cognate APC (nAPC) or a cognate APC (cAPC). The initial contact state is followed by a multi-state recognition process that terminates at state N. Transitions between states are reversible, with the exception of the final state at the end of the cAPC arm; here the T cell is immediately activated and can no longer transition back. We evaluate the overall activation probability and define the conditional moments of the activation time. From the latter, we calculate the mean and variance of the first time for a T cell to be activated by a cAPC under both Neumann and Robin boundary conditions at the spherical boundary representing the T cell zone. In section 4 we consider an alternative scenario where a T cell can fully bind to both nAPCs and cAPCs once their respective multi-stage chains have been traversed. However, intermediate states in each chain can “reset”, returning the T cell to its initial state of engagement with the nAPC or cAPC. This recycling represents a kinetic proofreading mechanism that can increase sensitivity to kinetic parameters, allowing for higher specificity.

## 3. Reversible multi-stage two-arm model.

We assume recognition involves T cells engaging with APCs through multiple interaction steps that lead to full activation only in the case of cAPCs. Similar models have been used to study viral entry into cells [8,17]. A schematic of the model with N intermediate states between a T cell and either APC is shown in (3.1). Here, the free T cell, denoted by ${\text{T}}_{0}$, can bind to a nAPC at rate $K_{\text{n}}$ to form the first bound state ${\text{N}}_{1}$, or with a cAPC at rate $fK_{\text{n}}$ to form the first bound state ${\text{C}}_{1}$. Microscopically, $K_{\text{n}}=K_{0,1}N$ and $K_{\text{c}}=K_{0,1}C$, where $K_{0,1}$ is an intrinsic attachment prefactor, and N and C denote the concentrations of available nAPCs and cAPCs, respectively. Rewriting $K_{\text{c}}=(C/N)K_{\text{n}}\equiv fK_{\text{n}}$, the parameter f can be interpreted as the ratio of the two concentrations; since the concentration of nAPCs is much greater than that of cAPCs, f≪1. The unbinding rates are $K_{1,0}$ in both arms.


(3.1)

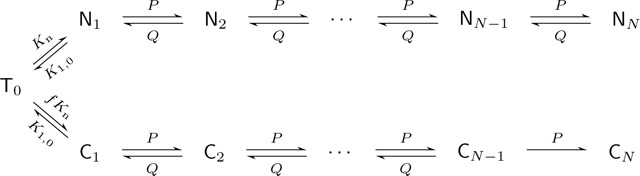



The nAPC interaction chain, shown in the upper arm of the scheme in (3.1) contains a sequence of intermediate bound states, ${\text{N}}_{2},\ldots {\text{N}}_{N-1}$, and is fully reversible, with the bidirectional forward and backward rates given by P and Q, respectively [23]. The reflecting boundary condition at the last ${\text{N}}_{N}$ state represents a “dead-end” of the interaction between a T cell and the nAPC, where no further processing is triggered. The cAPC interaction chain is modeled through a similar pathway with N steps as shown in the lower arm of (3.1). Here, the absorbing state ${\text{C}}_{N}$ represents a T cell that is activated by its cAPC. For simplicity, we assume uniform binding and unbinding rates P, Q and compute the probability of activation and the activation time statistics.

### 3.1. Diffusion-kinetic equations and non-dimensionalization.

We now quantify the dynamics of the system, including motion in the T cell zone and the kinetics depicted in (3.1). Since searcher T cells have no prior information on the location of APCs within the lymph node [12,46], we model them as three-dimensional Brownian walkers and assume their motion is arrested upon contact with an APC. We introduce x and t as the spatial and temporal variables, and denote the probability density distribution of finding a T cell at location x at time t as $\rho_{0}(x,t)$. We further denote the probability density distribution of a T cell bound to a nAPC or to a cAPC, respectively, via the N-dimensional vectors n(x,t) and c(x,t). The corresponding $n_{i}(x,t)$ and $c_{i}(x,t)$ components represent the probability of having a T cell bound to the nAPC or cAPC at the i-th state of engagement, with 1≤i≤N. T cell activation is triggered at state $c_{\text{N}}$.

Although the effective diffusion constant D(x) describing the motion of the T cells may be spatially dependent, we impose it to be uniform D(x)=D. Similarly, we assume $K_{0,1}$, $K_{1,0}$, N, C are spatially homogeneous so that $K_{\text{n}}$ and f are uniform as well. Finally, we assume T cells exit from, or degrade within the T cell zone at rate $\mu_{0}$. The above assumptions yield the following diffusion-kinetic equations

(3.2a) $$\partial_{t}\rho_{0}(x,t)=D\Delta \rho_{0}(x,t)-\left[\mu_{0}+(1+f)K_{0,1}\right]\rho_{0}+K_{1,0}\left(n_{1}+c_{1}\right),$$

(3.2b) $$\partial_{t}n(x,t)=M_{n}n+K_{0,1}\rho_{0}e_{1},$$

(3.2c) $$\partial_{t}c(x,t)=M_{c}c+fK_{0,1}\rho_{0},e_{1}.$$

where $e_{1}={\left(1,0,\ldots ,0\right)}^{T}\in {\mathbb{R}}^{N}$ and $M_{\text{n}}$ and $M_{\text{c}}$ are N×N tridiagonal matrices consistent with the reaction schemes shown in (3.1). For simplicity, we describe their elements in mathematical detail after first non-dimensionalizing (3.2). We model the T cell zone as a spherical domain Ω of radius a and impose Robin boundary conditions

(3.3) $$Dn\cdot \nabla \rho_{0}+K\rho_{0}=0,\;\text{on}\;\partial \text{Ω}.$$

The quantity K>0 in (3.3) represents the convective velocity at the boundary. The limit K→0 leads to the perfectly reflecting, Neumann boundary condition $n\cdot \nabla \rho_{0}=0$, where all traversing T cells are reflected to the interior. The opposite limit K→∞ leads to the perfectly absorbing, Dirichlet boundary condition $\rho_{0}=0$ which represents all T cells leaving the domain upon reaching its boundary. The partially absorbing, Robin boundary condition corresponds to a finite K and interpolates between the two limits. Finally, we utilize the initial condition

(3.4) $$\rho_{0}(x,0)=\delta (x),\;n(x,0)=0,\;c(x,0)=0,$$

so that at t=0 there are no bound APCs, and T cells are located at the center of the lymph node.

To non-dimensionalize (3.2)–(3.4) we define distances in terms of the radius of the T cell zone a and time in terms of the T cell – nAPC detachment time $1/K_{1,0}$. The dimensionless variables are thus defined as

(3.5) $$\begin{array}{l} \tilde{t}=K_{1,0}t,\;\tilde{x}=\frac{x}{a},\;{\tilde{\mu }}_{0}=\frac{\mu_{0}}{K_{1,0}},\;p=\frac{P}{K_{1,0}},\;q=\frac{Q}{K_{1,0}},\\ \tilde{D}=\frac{D}{a^{2}K_{1,0}},\;k_{0,1}=\frac{K_{\text{n}}}{K_{1,0}},\;\tilde{K}=\frac{K}{aK_{1,0}},\;\kappa =\frac{aK}{D}. \end{array}$$

Using estimates available from the literature, we set a=0.1cm, $K_{1,0}=1/3{\text{min}}^{-1}$, $D=60\mu {\text{m}}^{2}\min^{-1}$, and $\mu_{0}=1/720{\text{min}}^{-1}$. These values correspond to ${\tilde{\mu }}_{0}=1/240$ and $\tilde{D}=1.8\times 10^{-4}$. Parameter estimates are discussed in detail in Appendix A. For notational simplicity, we henceforth drop the tilde notation and find the dimensionless entries of $M_{n}$

(3.6) $${\left[M_{\text{n}}\right]}_{i,i}=\left\{\begin{array}{ll} -(p+1) & i=1\\ -(p+q) & 2\leq i\leq N-1\\ -q & i=N \end{array}\right.\;\begin{array}{ll} {\left[M_{\text{n}}\right]}_{i,i-1}=p & 2\leq i\leq N,\\ {\left[M_{\text{n}}\right]}_{i,i+1}=q & 1\leq i\leq N-1, \end{array}$$

while those for $M_{c}$ are

(3.7) $${\left[M_{\text{c}}\right]}_{i,i}=\left\{\begin{array}{ll} -(p+1) & i=1\\ -(p+q) & 2\leq i\leq N-1\\ 0 & i=N \end{array}\right.\;\begin{array}{l} {\left[M_{\text{c}}\right]}_{i,i-1}=p\;2\leq i\leq N\\ {\left[M_{\text{c}}\right]}_{i,i+1}=\left\{\begin{array}{l} q\;1\leq i\leq N-2\\ 0\;i=N-1. \end{array}\right. \end{array}$$

$M_{n}$ and $M_{c}$ differ only in that the last, activated state at the end of the cAPC chain is absorbing, while the end-state of the nAPC chain is reflecting. The full non-dimensional model is defined in the three-dimensional ball of unit radius $\text{Ω}=B_{1}^{3}(0)$:

(3.8a) $$\partial_{t}\rho_{0}(x,t)=D\Delta \rho_{0}(x,t)-\left[\mu_{0}+(1+f)k_{0,1}\right]\rho_{0}+n_{1}+c_{1},$$

(3.8b) $$\partial_{t}n(x,t)=M_{n}n+k_{0,1}\rho_{0}e_{1},$$

(3.8c) $$\partial_{t}c(x,t)=M_{c}c+fk_{0,1}\rho_{0},e_{1},$$

with boundary condition

(3.9) $$n\cdot \nabla \rho_{0}+\kappa \rho_{0}=0,\;\text{on}\;\partial \text{Ω}.$$

We now define the overall activation flux $J_{*}(t)$

(3.10) $$J_{*}(t):=\int_{\text{Ω}}\partial_{t}c_{N}(x,t)\text{d}x=\int_{\text{Ω}}pc_{N-1}(x,t)\text{d}x,$$

and the activation probability

(3.11) $$P_{*}:=\int_{0}^{\infty }J_{*}(t)\text{d}t,$$

from which the conditional activation flux $J_{*,\text{c}}(t)=J_{*}(t)/P_{*}$ is derived [9]. Finally, the conditional moments $E\left[{\left(T_{*}\right)}^{h}\right]$ of the activation time are

(3.12) $$E\left[{\left(T_{*}\right)}^{h}\right]:=\int_{0}^{\infty }t^{h}J_{*,\text{c}}(t)\text{d}t=\frac{\int_{0}^{\infty }t^{h}J_{*}(t)\text{d}t}{\int_{0}^{\infty }J_{*}(t)\text{d}t}.\;h\geq 0.$$

The conditional mean activation time $\tau_{*}$ and variance $\sigma_{*}^{2}$ are obtained by substituting h=1 and h=2 into (3.12), respectively,

(3.13) $$\tau_{*}=E\left(T_{*}\right),\;\sigma_{*}^{2}=E\left[\left[{\left(T_{*}\right)}^{2}\right]-\tau_{*}^{2}.\right.$$

The time $\tau_{\text{nAPC}}$ that a T cell spends engaged with a nAPC before detaching and resuming its search for a cAPC can be estimated by calculating the average time it takes a T cell starting in state ${\text{N}}_{1}$ to first reach the free state ${\text{T}}_{0}$. As depicted in (3.1), the T cell performs a random walk across the N nAPC bound states detaching once ${\text{T}}_{0}$ is reached. In section C, we show the non-dimensional $\tau_{\text{nAPC}}$ is

(3.14) $$\tau_{\text{nAPC}}=\frac{1-{\left(p/q\right)}^{N}}{1-p/q}.$$

The interaction time in (3.14) is an increasing function of N, regardless of p/q, and an increasing function of p/q, regardless of N. Thus, the more intermediate states there are along the nAPC chain, the longer the T cell remains unproductively engaged. Similarly, the larger the ratio of the forward to backward rates p/q, the longer it will take for the T cell to return to the free state.

### 3.2. Neumann (perfectly reflecting) boundary conditions.

We begin our analysis under perfectly reflecting, Neumann boundary conditions by setting κ=0 in (3.9), and calculate the activation probability $P_{*}$ in (3.11), and the conditional mean activation time $\tau_{*}$ in (3.13). For an arbitrary function y(x,t), x∈Ω we denote $\bar{y}(t)=\int_{\text{Ω}}y(x,t)dx$. By the Divergence Theorem, we have

(3.15) $$\int_{\text{Ω}}\text{Δ}\rho_{0}dx=\int_{\partial \text{Ω}}\partial_{n}\rho_{0}\;ds=0,$$

where the left equality is due to the Neumann boundary condition for $\rho_{0}$ in (3.9). Using (3.15) and upon integrating each equation in (3.8) over the entire domain Ω, we obtain the following ODE system

(3.16) $$\begin{array}{l} \frac{\text{d}\bar{y}(t)}{\text{d}t}=M\bar{y}(t),\;\bar{y}(t)={\left({\bar{\rho }}_{0}(t),\bar{n}(t),\bar{c}(t)\right)}^{T},\\ \;\bar{y}(0)=(1,0,\cdots ,0)\in {\mathbb{R}}^{2N+1}, \end{array}$$

where $\bar{n}(t)\equiv \left({\bar{n}}_{1}(t),\ldots ,{\bar{n}}_{N}(t)\right)$ and $\bar{c}(t)\equiv \left({\bar{c}}_{1}(t),\ldots ,{\bar{c}}_{N}(t)\right)$. The matrix M is given by

(3.17) $$M=\left[\begin{array}{ccc} -\left[\mu_{0}+(1+f)k_{0,1}\right] & e_{1}^{T} & e_{1}^{T}\\ k_{0,1}e_{1} & M_{\text{n}} & O\\ fk_{0,1}e_{1} & O & M_{\text{c}} \end{array}\right]$$

where O denotes the N×N zero matrix, and $M_{\text{n}}$ and $M_{\text{c}}$ are the N×N tridiagonal matrices defined in (3.6) and (3.7), respectively. In this construction, M is a (2N+1)×(2N+1) matrix. Eq. (3.16) can be solved by applying the Laplace transform to Eq. (3.16) with the nonabsorbed states excluded (omitting ${\bar{c}}_{N}$ since it can be determined from ${\bar{c}}_{N-1}$). Upon defining the N−1 dimensional vector ${\bar{c}}^{\prime }(s)={\left({\bar{c}}_{1}(s),\ldots ,{\bar{c}}_{N-1}(s)\right)}^{T}$ and taking the Laplace transform of the truncated linear system (3.16) that excludes ${\bar{c}}_{N}$, we find

(3.18) $$s{\bar{y}}^{\prime }(s)-{\bar{y}}^{\prime }(t=0)=M^{\prime }{\bar{y}}^{\prime }(s),\;{\bar{y}}^{\prime }(s)={\left({\bar{\rho }}_{0}(s),\bar{n}(s),{\bar{c}}^{\prime }(s)\right)}^{T},$$

where $M^{\prime }$ is the same as M without the last row and last column, and thus a 2N×2N matrix. The formal solution to Eq. (3.18) is

(3.19) $${\bar{y}}^{\prime }(s)={\left(sI-M^{\prime }\right)}^{-1}{\bar{y}}^{\prime }(t=0).$$

Differentiating (3.18) with respect to s and then setting s=0 gives

(3.20) $${\left.\frac{\text{d}{\bar{y}}^{\prime }(s)}{\text{d}s}\right|}_{s=0}={\left(M^{\prime }\right)}^{-1}{\bar{y}}^{\prime }(s=0).$$

Eqs. (3.19) and (3.20) allow us to compute the activation probability $P_{*}$ and the conditional mean activation time $\tau_{*}$, both of which can be expressed in terms of the Laplace variable through (3.10) to (3.13) as

(3.21) $$P_{*}=p{\bar{c}}_{N-1}(s=0),$$

(3.22) $$\tau_{*}=-{\left.\frac{1}{{\bar{c}}_{N-1}(s=0)}\frac{\text{d}{\bar{c}}_{N-1}(s)}{\text{d}s}\right|}_{s=0}.$$

Upon setting s=0 in (3.19) and inverting $M^{\prime }$, we obtain ${\bar{y}}^{\prime }(s=0)$; its last entry, ${\bar{c}}_{N-1}(s=0)$, can be inserted into (3.21) to obtain

(3.23) $$P_{*}=\frac{fk_{0,1}(p-q)}{fk_{0,1}(p-q)+\mu_{0}\left[p-q+1-{\left(\frac{q}{p}\right)}^{N-1}\right]}.$$

We similarly evaluate $\text{d}{\bar{y}}^{\prime }(s)/\text{d}s$ for s=0 from Eq. (3.20); its last entry and ${\bar{c}}_{N-1}(s=0)$ are then substituted into Eq. (3.22) to obtain $\tau_{*}$.

**Fig. 2:**
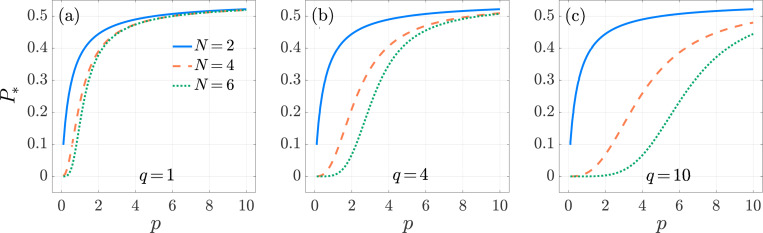
Multi-stage, Neumann boundary conditions. The activation probability $P_{*}$ as a function of the forward binding rate p in the multi-stage model (3.8) for N=2 (blue-solid), 4, (orange-dashed), and 6 (green-dotted). We set $f=5\times 10^{-3}$, $k_{0,1}=1$, $\mu_{0}=1/240$ and q=1 in panel (a), q=4 in panel (b) and q=10 in panel (c). The values of $P_{*}$ are computed from (3.23) All curves are monotonically increasing.

Results are shown in Fig. 2 for various choices of q and N=2,4,6. The activation likelihood $P_{*}$ is a competition between degradation, expressed by $\mu_{0}$, and reaching the final state of the cAPC arm. The schematic (3.1) reveals that for N=2 the detachment rate q affects the dynamics only as a T cell engages with a nAPC. However, since the nAPC arm is characterized by reflecting boundary conditions at its end state and no degradation is present, we expect $P_{*}$ to be independent of q for N=2. This is observed in Fig. 2, where $P_{*}$ is also seen to be increasing in p for N=2.

Higher values of p result in a higher likelihood that T cells remains partially engaged to their cAPCs or to the other nAPCs, rather than detaching from the first bound state, as can be seen by the form ${\left[M_{n}\right]}_{ii}={\left[M_{c}\right]}_{ii}=-(p+1)$. In our model, excursions along the nAPC arm do not increase the likelihood of degradation, rather they shield T cells from the degradation that they would experience in the unbound state ${\text{T}}_{0}$. At the same time, higher p values allow for T cells to be fully activated by their cAPC by reaching state ${\text{C}}_{N}$ in a more expedited manner, escaping degradation while in the free state. The overall effect of increasing p is thus to increase $P_{*}$. Since this picture remains valid for other choices N>2, the finding that $P_{*}$ increases with p should be robust with respect to changes in q and N. To understand how $P_{*}$ depends on q (for N>2) and on N, we note that as q increases the likelihood of returning from any intermediate state ${\text{N}}_{i}$ or ${\text{C}}_{j}$ with i, j<N to the unbound state ${\text{T}}_{0}$, and thus for T cells to be degraded, increases. Hence, $P_{*}$ should decrease with q for fixed p and N>2. Similarly, we expect $P_{*}$ to decrease with increasing N since the presence of more intervening steps to reach the activation state ${\text{C}}_{N}$ is associated with a greater likelihood for the T cell to return to the free state ${\text{T}}_{0}$, where it is subject to degradation. The curves shown in Fig. 2 confirm that $P_{*}$ is an increasing function of p and a decreasing function of q and N when all other quantities are kept fixed. Finally, we verified that for all cases shown in Fig. 2, increasing the degradation $\mu_{0}$ decreases $P_{*}$ as expected.

**Fig. 3:**
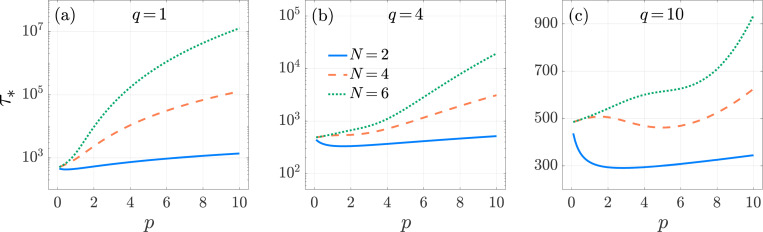
Multi-stage, Neumann boundary conditions. The conditional mean activation time $\tau_{*}$ as a function of the forward binding rate p in the multi-stage model (3.8) for N=2 (blue-solid), 4 (orange-dashed), and 6 (green-dotted). We set $f=5\times 10^{-3}$, $k_{0,1}=1$, $\mu_{0}=1/240$ and q=1 in panel (a), q=4 in panel (b) and q=10 in panel (c). The values of $\tau_{*}$ are computed from (3.22) and (3.20). Depending on parameter choices, $\tau_{*}$ can exhibit non-monotonic behavior.

In Fig. 3 we plot $\tau_{*}$ as a function of p for various values of N and q. We observe that $\tau_{*}$ exhibits non-monotonic behavior as a function of p. This is because, on one hand, increasing p elongates the time a T cell remains on the nAPC arm, increasing $\tau_{*}$; on the other, it hastens the time for the T cell to be fully activated at the end of the cAPC arm, decreasing $\tau_{*}$. Which of these trends prevail depends on the interplay between the magnitude of p, q and the length N of the nAPC and cAPC arms. This is illustrated by setting N=2, in which case $P_{*}$ and $\tau_{*}$ can be evaluated explicitly

(3.24) $$P_{*}=\frac{fk_{0,1}p}{fk_{0,1}p+\mu_{0}(p+1)},\;\text{for}\;N=2$$

(3.25) $$\tau_{*}=\frac{\left(1+\mu_{0}+p\right)q+k_{0,1}(p+q)(p+1)+fk_{0,1}q}{q\left[fk_{0,1}p+\mu_{0}(p+1)\right]},\;\text{for}\;N=2.$$

Eqs. (3.24) and (3.25) show that $P_{*}$ monotonically increases with p but that $\tau_{*}$, can be non-monotonic in p depending on the other parameters. One can evaluate the loci of the minima in $\tau_{*}$ as a function of q by taking the derivative of (3.25) with respect to p. We find that, as a function of p, $\tau_{*}$ displays a minimum only if $q>q^{*}$ where

(3.26) $$q^{*}=\frac{fk_{0,1}^{2}\mu_{0}}{\left(fk_{0,1}+\mu_{0}\right)\left[fk_{0,1}\left(1+k_{0,1}+fk_{0,1}\right)+2fk_{0,1}\mu_{0}+\mu_{0}^{2}\right]}<1.$$

Non-monotonic behavior is observed in Fig. 3 for N>2 as well. Here, $\tau_{*}$ is seen to increase with p, except for intermediate p values where a decreasing trend emerges. The decreasing regimes correspond to optimal p ranges where the T cell can be activated by reaching the end of the cAPC arm while shortening excursion times along the nAPC arm. Although general analytical estimates are not possible, we observe that the values of p that minimize $\tau_{*}$ tend to increase with N. The expression in (3.25) also reveals that for fixed p, $\tau_{*}$ is a decreasing function of q for N=2. Fig.3 shows that $\tau_{*}$ decreases with q also for N>2. Larger values of q diminish the time a T cell spends engaged with a nAPC while having no effect on the time spent with its cAPC, so that larger q should lead to lower $\tau_{*}$, as observed. Fig. 3 also shows that $\tau_{*}$ increases with N. In this case, increasing the length N of the cAPC arm, results in the T cell requiring a longer time to reach the final activation stage.

Finally, we expect $\tau_{*}$ to decrease as f or $\mu_{0}$ is increased. Increasing f increases the likelihood that the T cell encounters its cAPC, shortening the time to full activation. Since increasing $\mu_{0}$ hastens the degradation process, the conditional mean activation time must be shorter to avoid degradation. Both of these trends in $\tau_{*}$ are observed for all p, q, N values surveyed.

### 3.3. Extreme first activation time statistics.

The results in section 3.2 for the activation flux $J_{*}$, activation probability $P_{*}$, and conditional mean activation time $\tau_{*}$ hold for a single T cell released at x=0 at t=0. However, if the process is initialized with a collection of m T cells at the origin, one may wish to evaluate the probability and first activation time of any T cell. Provided m is not too large as to significantly deplete the pool of free APCs, the T cells can be considered independent particles. For any specific T cell, we compute the conditional survival probability $S_{\text{c}}(t)$, defined as the probability the T cell has not activated up to time t, given that it will activate. We do so by explicitly solving $\frac{\text{d}S_{\text{c}}(t)}{\text{d}t}=-J_{*,\text{c}}(t)$ with the initial condition $S_{\text{c}}(0)=1$ so that $S_{\text{c}}(t)=1-\int_{0}^{t}J_{*,\text{c}}\left(t^{\prime }\right)\text{d}t^{\prime }$. From this quantity, we construct moments of the first activation time of *any* T cell. First, we define the probability that the minimum activation time $T_{\min}$ among all m initial T cells occurs after time t

(3.27) $$\mathbb{P}\left(T_{\min}>t\right):=S_{\min}(t)=\left[{\left(1-P_{*}\left(1-S_{\text{c}}(t)\right)\right]}^{m}\right.$$

The term $P_{*}\left(1-S_{\text{c}}(t)\right)$ represents the probability that a T cell has activated, and has done so by time t. $S_{\min}(t)$ is the overall survival function, the probability that none of the m available T cells has been activated up to time t, regardless of whether they will eventually be degraded, exit the T cell zone, or activate.

We now subtract the probability that none of the m T cells activate from (3.27) the probability that none of the m T cells activate, due to degradation or egress from the T cell zone, given by ${\left(1-P_{*}\right)}^{m}$.

The difference is the probability that $T_{\min}>t$ and at least one T cell activates. This difference is also the probability that $T_{\min}>t$ *conditioned on* at least one T cell activating, times the probability $P_{*}^{\left(m\right)}$ that at least one T cell activates. Since T cells are independent, $P_{*}^{\left(m\right)}=1-{\left(1-P_{*}\right)}^{m}$ and

(3.28) $$\begin{array}{l} \mathbb{P}\left(T_{\min}<t|T_{\min}<\infty \right):=S_{\min}\left(t|T_{\min}<\infty \right)\\ \;=\frac{\left[{\left(1-P_{*}\left(1-S_{\text{c}}(t)\right)\right]}^{m}-{\left(1-P_{*}\right)}^{m}\right.}{1-{\left(1-P_{*}\right)}^{m}}. \end{array}$$

We now define the conditional first activation time distribution $w_{\min}\left(t|T_{\min}<\infty \right)=-\frac{\text{d}S_{\min}\left(t|T_{\min}<\infty \right)}{\text{d}t}$ and use it to compute moments of the first activation time

(3.29) $$E\left[T_{\min}^{h}\right]=\int_{0}^{\infty }t^{h}w_{\min}\left(t|T_{\min}<\infty \right)\text{d}t,$$

and the corresponding standard deviation

(3.30) $$\sigma_{\min}:=\sqrt{E\left[{\left(T_{\min}\right)}^{2}\right]-E\left[T_{\min}\right]}.$$

**Fig. 4:**
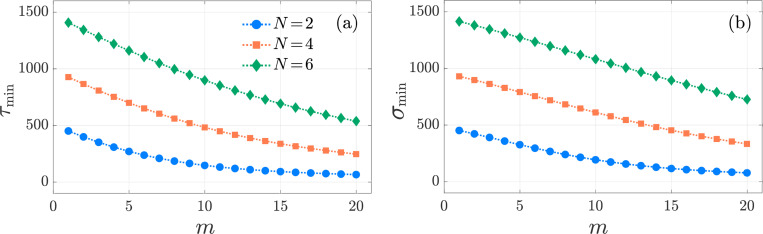
Multi-stage, Neumann boundary conditions. The conditional mean and standard deviation of the minimum activation time of m T cells in the multi-stage model (3.8) for N=2 (blue-circle), N=4 (orange-square), and N=6 (greendiamond). We set $f=5\times 10^{-3}$, $k_{0,1}=1$, $\mu_{0}=1/240$, p=q=1. The values of $\tau_{\min}$ and $\sigma_{\min}$ are computed from (3.29) for h=1, and (3.30), respectively. All curves are monotonically decreasing.

In Fig. 4 we plot the conditional mean $\tau_{\min}:=E\left[T_{\min}\right]$ and the standard deviation $\sigma_{\min}$. As expected, $E\left[T_{\min}\right]$ decreases with the number of searcher T cells m. The corresponding standard deviation, $\sigma_{\min}$, also decreases with m and remains comparable in magnitude to the mean. This relationship suggests that a Poisson distribution can reasonably approximate the conditional first activation time distribution.

### 3.4. Robin (partially reflecting) boundary conditions.

We now explore how $P_{*}$, $\tau_{*}$ vary under Robin boundary conditions, for finite κ in (3.9). Since in this case it is not possible to reduce (3.8) to a series of coupled ODEs, we must retain the inherent spatial dependence. Thus, we first write the formal time-dependent solution to (3.8b) and (3.8c). In Appendix D, we show that the kinetic matrices $M_{\text{n}}$ and $M_{\text{c}}$ are diagonalizable and can be written as

(3.31) $$M_{\text{n}}=V_{\text{n}}\Lambda_{\text{n}}V_{\text{n}}^{-1},\;M_{\text{c}}=V_{\text{c}}\Lambda_{\text{c}}V_{\text{c}}^{-1},$$

where $V_{\text{n}}$ and $V_{\text{c}}$ are N×N matrices whose columns consist of the eigenvectors of $M_{\text{n}}$ and $M_{\text{c}}$, respectively. The diagonal matrices $\Lambda_{\text{n}}=\mathrm{diag}\left(\lambda_{\text{n}}^{1},\lambda_{\text{n}}^{2},\ldots ,\lambda_{\text{n}}^{N}\right)$ and $\Lambda_{\text{c}}=\mathrm{diag}\left(\lambda_{\text{c}}^{1},\lambda_{\text{c}}^{2},\ldots ,\lambda_{\text{c}}^{N}\right)$ consist of the corresponding eigenvalues of $M_{\text{n}}$ and $M_{\text{c}}$ respectively. Using the decompositions in (3.31) the solution components of (3.8b) and (3.8c) can be written as

(3.32) $$\begin{array}{l} n_{i}(x,t)=k_{0,1}\sum_{j=1}^{N}{\left[V_{\text{n}}\right]}_{i,j}{\left[V_{\text{n}}^{-1}\right]}_{j,1}\int_{0}^{t}e^{\lambda_{\text{n}}^{j}\left(t-t^{\prime }\right)}\rho_{0}\left(x,t^{\prime }\right)\text{d}t^{\prime },\;1\leq i\leq N,\\ c_{i}(x,t)=fk_{0,1}\sum_{j=1}^{N}{\left[V_{\text{c}}\right]}_{i,j}{\left[V_{\text{c}}^{-1}\right]}_{j,1}\int_{0}^{t}e^{\lambda_{\text{c}}^{j}\left(t-t^{\prime }\right)}\rho_{0}\left(x,t^{\prime }\right)\text{d}t^{\prime },\;1\leq i\leq N. \end{array}$$

By substituting the explicit expressions of $n_{1}$ and $c_{1}$ from (3.32) into (3.8a), we obtain the integro-differential equation (IDE)

(3.33) $$\partial_{t}\rho_{0}=D\Delta \rho_{0}-\left[\mu_{0}+(1+f)k_{0,1}\right]\rho_{0}+\int_{0}^{t}K\left(t-t^{\prime }\right)\rho_{0}\left(x,t^{\prime }\right)\text{d}t^{\prime },$$

where the memory kernel K(t) is defined by

(3.34) $$K(t)=k_{0,1}\sum_{j=1}^{N}{\left[V_{\text{n}}\right]}_{1,j}{\left[V_{\text{n}}^{-1}\right]}_{j,1}e^{\lambda_{\text{n}}^{j}t}+fk_{0,1}\sum_{j=1}^{N}{\left[V_{c}\right]}_{1,j}{\left[V_{c}^{-1}\right]}_{j,1}e^{\lambda_{\text{c}}^{j}t}.$$

We now use separation of variables as illustrated in section B to derive $\rho_{0}(x,t)$ under the Robin boundary condition in (3.9). This quantity can then be used to determine $n_{i}(x,t)$ and $c_{i}(x,t)$ for i=1,2,…,N from (3.32), finally calculating $P_{*}$ and $\tau_{*}$ via (3.10) to (3.13).

**Fig. 5:**
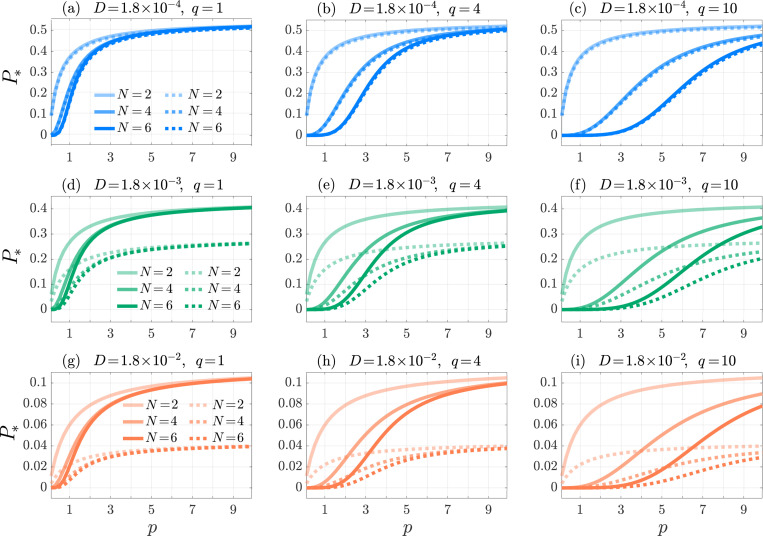
Multi-stage, Robin boundary conditions. The activation probability $P_{*}$ as a function of the forward binding rate p in the multi-stage model (3.8) for N=2,4,6 (from lighter to darker shades) and Robin coefficient κ=1 (solid) and κ→∞ (dotted). We set $f=5\times 10^{-3}$, $k_{0,1}=1$, $\mu_{0}=1/240$, and $D=1.8\times 10^{-4}$ (top row, blue), 1.8×10^−3^ (middle row, green), 1.8×10^−2^ (bottom row, orange), and q=1,4,10 (from left to right). The values of $P_{*}$ are computed from (3.11), (3.10), (3.32). All curves are monotonically increasing.

Results are shown in Fig. 5 where the activation probability $P_{*}$ is plotted as a function of p for various values of q, N, κ, D. Trends that were observed under the perfectly reflecting, Neumann boundary conditions in Fig. 2 and that correspond to κ=0, are the same. For example, $P_{*}$ remains an increasing function of p and a decreasing function of q and N when all other quantities are kept fixed, mirroring the results in Fig. 2. The main difference is that under Robin boundary conditions there is an extra dependence on κ and D. First, we note that increasing κ should decrease the activation probability $P_{*}$ since a larger κ implies a higher likelihood that the T cell leaves the T cell zone before activation by its cAPC. We expect that increasing the diffusion constant D should also decrease $P_{*}$ since larger D is equivalent to favoring transport over binding to any APC. This, in turn, would imply that the T cell is more likely to be exposed to degradation, since the latter is only experienced in the free form and not when the T cell is bound to any APC. That $P_{*}$ should decrease with D can be also be easily verified from the explicit solution for $P_{*}$ in (B.15) of section B. Fig. 5 confirms that $P_{*}$ decreases as κ and D increase. As in Fig. 2, increasing the degradation rate $\mu_{0}$ decreases $P_{*}$ under Robin boundary conditions as well.

**Fig. 6:**
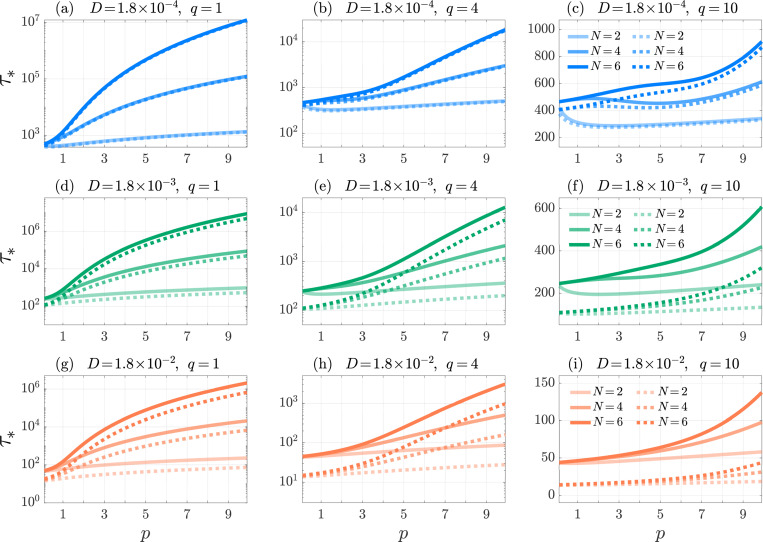
Multi-stage, Robin boundary conditions. The conditional mean activation time $\tau_{*}$ as a function of the forward binding rate p in the multi-stage model (3.8) for N=2,4,6 (from lighter to darker shades) and Robin coefficient κ=1 (solid) and κ→∞ (dotted). We set $f=5\times 10^{-3}$, $k_{0,1}=1$, $\mu_{0}=1/240$, and $D=1.8\times 10^{-4}$ (top row, blue), 1.8 × 10^−3^ (middle row, green), 1.8 × 10^−2^ (bottom row, orange), and q=1,4,10 (from left to right). The values of $\tau_{*}$ are computed from (3.12) for h=1, (3.10), (3.32). Depending on parameter choices, $\tau_{*}$ can exhibit non-monotonic behavior.

Since increasing κ or D leads to faster escape, we expect the conditional mean activation time $\tau_{*}$ to decrease with κ and D. This dependence is shown in Fig. 6 which plots $\tau_{*}$ as a function of p for various values of q, N, κ, D. As in Fig. 3, we observe that $\tau_{*}$ remains a non-monotonic function of p, a decreasing function of q, and an increasing function of N when all other quantities are kept fixed.

## 4. Kinetic proofreading.

In the previous section, we assumed that the cAPC and nAPC multi-stage arms are similar in that reactions proceed forward (with rate p) or backward (with rate q) within both. At the end of the N-state chain the T cell either binds irreversibly to its cAPC (activates), or reaches an nAPC dead-end, as shown in (3.1). Here, we modify the previous scheme in two ways. First, we allow for T cells to completely disengage from any of the intermediate steps along any APC chain to return to the free state. Thus, when modeling interactions between T cells and cAPCs, we replace the incremental backward step from ${\text{C}}_{i}$ to ${\text{C}}_{i-1}$ with a return step from ${\text{C}}_{i}$ to ${\text{T}}_{0}$. A similar modification is applied for steps along nAPC arm. Second, we assume that T cells can irreversibly bind to nAPCs at the end of the nAPC arm as well, so that both end-states along the cAPC and nAPC arms are absorbing. This is a canonical reaction scheme that supports “kinetic proofreading” and is illustrated in (4.1) and (4.2). Kinetic proofreading (KPR) was first introduced in the 1970s to offer a paradigm that could explain low error rates in DNA replication [22,42]. Later, it was applied to study the high specificity of T cell receptors in recognizing cAPCs [31,32,36]. Here, we invoke the KPR mechanism to evaluate how the T cell enhances its selection of the cAPC over nAPCs.


(4.1)

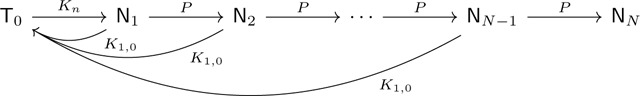




(4.2)

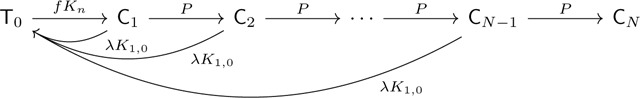



In dimensional units, the rate at which the T cell in any state ${\text{N}}_{i}$ disassembles and “resets” to the free state is denoted $K_{1,0}$, along the nAPC arm. The corresponding disassembly or reseting rates are $\lambda K_{1,0}$ along the cAPC arm. We assume that cAPC complexes are modestly more stable than nAPC complexes so that their disassembly rates are slower, λ<1. Because nAPCs are more abundant than cAPCs (f≪1), T cells are kinetically more likely to bind to nAPCs. We utilize (3.5) to nondimensionalize the return rates $K_{0,1}$ and $\lambda K_{0,1}$ to 1 and λ, respectively. The non-dimensional kinetics of the two-arm resetting model depicted in (4.1) and (4.2) are described by

(4.3a) $$\partial_{t}\rho_{0}(x,t)=D\Delta \rho_{0}(x,t)-\left[\mu_{0}+(1+f)k_{0,1}\right]\rho_{0}+\sum_{i=1}^{N-1}n_{i}+\lambda \sum_{i=1}^{N-1}c_{i},$$

(4.3b) $$\partial_{t}n(x,t)=B_{\text{n}}n+k_{0,1}\rho_{0}e_{1},$$

(4.3c) $$\partial_{t}c(x,t)=B_{\text{c}}c+fk_{0,1}\rho_{0}e_{1}.$$

The N×N bidiagonal matrices $B_{\text{n}}$ and $B_{\text{c}}$ describe interactions between T cells and APCs and incorporate disassembly and adsorption at the last stage of the N-state chains. The non-dimensional entries of $B_{\text{n}}$ are

(4.4) $${\left[B_{\text{n}}\right]}_{i,i}=\left\{\begin{array}{ll} -(1+p) & 1\leq i\leq N-1,\\ 0 & i=N, \end{array}\;{\left[B_{\text{n}}\right]}_{i,i-1}=p\;2\leq i\leq N-1,\right.$$

whereas those pertaining to $B_{\text{c}}$ are

(4.5) $${\left[B_{\text{c}}\right]}_{i,i}=\left\{\begin{array}{ll} -(\lambda +p) & 1\leq i\leq N-1,\\ 0 & i=N, \end{array}\;{\left[B_{\text{c}}\right]}_{i,i-1}=p\;2\leq i\leq N-1.\right.$$

Finally, we employ the previous initial and boundary conditions, Eqs. (3.4) and (3.9), respectively. We now define $P_{*,\text{n}}$ and $P_{*,\text{c}}$ as the probabilities that a T cell is “improperly” activated by an nAPC and “properly” activated by its cAPC, respectively. To evaluate these quantities, we first introduce the two fluxes into nAPC- and cAPC-induced activation states

(4.6a) $$J_{\text{n}}(t):=\int_{\text{Ω}}\partial_{t}n_{N}(x,t)dx=\int_{\text{Ω}}p\;n_{N-1}(x,t)dx,$$

(4.6b) $$J_{\text{c}}(t):=\int_{\text{Ω}}\partial_{t}c_{N}(x,t)dx=\int_{\text{Ω}}pc_{N-1}(x,t)dx.$$

The probabilities $P_{*,\text{n}}$ and $P_{*,\text{c}}$ are given by

(4.7) $$P_{*,\text{n}}:=\int_{0}^{\infty }J_{\text{n}}(t)\text{d}t,\;P_{*,\text{c}}:=\int_{0}^{\infty }J_{\text{c}}(t)\text{d}t.$$

leading to the two conditional fluxes into the activation states

(4.8) $$\begin{array}{l} J_{*,\text{n}}(t):=\frac{J_{\text{n}}(t)}{P_{*,\text{n}}}=\frac{1}{P_{*,\text{n}}}\int_{\text{Ω}}p\;n_{N-1}(x,t)\text{d}x,\\ J_{*,\text{c}}(t):=\frac{J_{\text{c}}(t)}{P_{*,\text{c}}}=\frac{1}{P_{*,\text{c}}}\int_{\text{Ω}}pc_{N-1}(x,t)\text{d}x. \end{array}$$

The associated moments of the conditional first activation time to a nAPC, $E\left[T_{*,\text{n}}^{h}\right]$, and to a cAPC, $E\left[T_{*,\text{c}}^{h}\right]$, respectively, are

(4.9) $$E\left[T_{*,\text{n}}^{h}\right]=\int_{0}^{\infty }t^{h}J_{*,\text{n}}(t)\text{d}t,\;E\left[T_{*,\text{c}}^{h}\right]=\int_{0}^{\infty }t^{h}J_{*,\text{c}}(t)\text{d}t,$$

and the conditional mean activation times are obtained by setting h=1 in (4.9),

(4.10) $$\tau_{*,\text{n}}:=E\left[T_{*,\text{n}}\right],\;\tau_{*,\text{c}}:=E\left[T_{*,\text{c}}\right].$$

Finally, we introduce the cAPC activation specificity $F_{\text{c}}$, defined as the activation likelihood of a T cell to a cAPC relative to the total activation likelihood

(4.11) $$F_{\text{c}}=\frac{P_{*,\text{c}}}{P_{*,\text{n}}+P_{*,\text{c}}}.$$

Note that $F_{\text{c}}=P_{*,\text{c}}$ if Neumann boundary conditions are applied and $\mu_{0}=0$ since in this case $P_{*,\text{n}}+P_{*,\text{c}}=1$. As $F_{\text{c}}$ is the probability of activation by a cAPC, given activation, we expect it to be independent of the degradation rate $\mu_{0}$.

### 4.1. Neumann (perfectly reflecting) boundary conditions.

Predictions of the KPR mechanism under perfectly reflecting, Neumann boundary conditions can be derived by setting κ=0 in (3.3). The T cell can either be activated by its cAPC, by an nAPC, or be degraded within the T cell zone. Upon integrating (4.3) over $\text{Ω}=B_{1}^{3}(0)$, akin to the procedure used to derive (3.16), we obtain

(4.12) $$\frac{\text{d}\bar{y}(t)}{\text{d}t}=B\bar{y}(t),\;\bar{y}(t)={\left({\bar{\rho }}_{0}(t),\bar{n}(t),\bar{c}(t)\right)}^{T},$$

where ${\bar{\rho }}_{0}$, $\bar{n}$, $\bar{c}$, and their initial conditions are defined in (3.16). The matrix B in (4.12) is given by

(4.13) $$B=\left[\begin{array}{ccc} -\left[\mu_{0}+(1+f)k_{0,1}\right] & {\left(1-e_{N}\right)}^{T} & \lambda {\left(1-e_{N}\right)}^{T}\\ k_{0,1}e_{1} & B_{\text{n}} & O\\ fk_{0,1}e_{1} & O & B_{\text{c}} \end{array}\right]$$

where O is the N×N zero matrix, and $B_{\text{n}}$ and $B_{\text{c}}$ are the N×N matrices defined in (4.4) and (4.5), respectively. The N-dimensional vectors 1, $e_{1}$ and $e_{N}$ are defined as $1={\left(1,1,\cdots ,1\right)}^{T}$, $e_{1}={\left(1,0,\cdots ,0\right)}^{T}$ and $e_{N}={\left(0,\cdots ,0,1\right)}^{T}\in {\mathbb{R}}^{N}$, respectively. We now eliminate the equations for ${\bar{n}}_{N}$ and ${\bar{c}}_{N}$ in the ODE system (4.12); upon taking the Laplace transform of the truncated set of equations, we find

(4.14) $$s{\bar{y}}^{\prime }(s)-{\bar{y}}^{\prime }(t=0)=B^{\prime }y^{\prime }(s),\;{\bar{y}}^{\prime }(s)={\left({\bar{\rho }}_{0}(s),{\bar{n}}^{\prime }(s),{\bar{c}}^{\prime }(s)\right)}^{T},$$

where $B^{\prime }$ is the (2N−1)×(2N−1) matrix constructed by eliminating the rows and columns of B corresponding to ${\bar{n}}_{N}$ and ${\bar{c}}_{N}$, respectively. These are the ${\left(N+1\right)}^{\text{th}}$ and ${\left(2N+1\right)}^{\text{th}}$ rows and columns of B. Similarly, ${\bar{n}}^{\prime }(s)$ and ${\bar{c}}^{\prime }(s)$ are the Laplace transforms of $\bar{n}(t)$ and $\bar{c}(t)$ without the ${\bar{n}}_{N}$, ${\bar{c}}_{N}$ entries. Note, that since states $c_{N}$ and $n_{N}$ are absorbing, the subsystem in (4.14) is complete. By evaluating the Laplace transforms we find

(4.15a) $$P_{*,\text{n}}=\frac{k_{0,1}{\left(\frac{p}{1+p}\right)}^{N-1}}{\mu_{0}+k_{0,1}{\left(\frac{p}{1+p}\right)}^{N-1}+fk_{0,1}{\left(\frac{p}{\lambda +p}\right)}^{N-1}},$$

(4.15b) $$P_{*,\text{c}}=\frac{fk_{0,1}{\left(\frac{p}{\lambda +p}\right)}^{N-1}}{\mu_{0}+k_{0,1}{\left(\frac{p}{1+p}\right)}^{N-1}+fk_{0,1}{\left(\frac{p}{\lambda +p}\right)}^{N-1}},$$

and

(4.16) $$\tau_{*,\text{n}}=\frac{N-1}{1+p}+T_{*},\;\tau_{*,\text{c}}=\frac{N-1}{\lambda +p}+T_{*},$$

where

$$T_{*}=\frac{1+k_{0,1}\left[1-{\left(\frac{p}{1+p}\right)}^{N-1}\left(1+\frac{N-1}{1+p}\right)\right]+fk_{0,1}\left[\frac{1}{\lambda }-{\left(\frac{p}{\lambda +p}\right)}^{N-1}\left(\frac{1}{\lambda }+\frac{N-1}{\lambda +p}\right)\right]}{\mu_{0}+k_{0,1}{\left(\frac{p}{1+p}\right)}^{N-1}+fk_{0,1}{\left(\frac{p}{\lambda +p}\right)}^{N-1}}.$$

The likelihood for a T cell to be degraded is $1-P_{*,\text{c}}-P_{*,\text{n}}$ and, as can be seen from (4.15a) and (4.15b), is zero if $\mu_{0}=0$. Upon substituting (4.15) into (4.11), it can be verified that the activation specificity is

(4.17) $$F_{\text{c}}=\frac{f}{f+{\left(\frac{\lambda +p}{1+p}\right)}^{N-1}}.$$

**Fig. 7:**
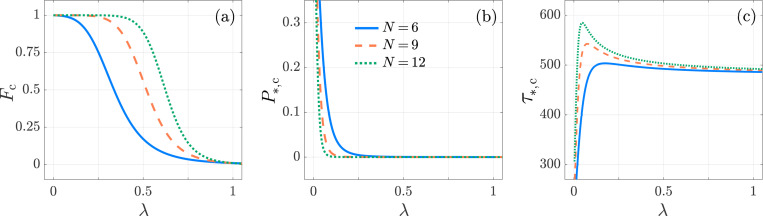
Kinetic proofreading, Neumann boundary conditions. The activation specificity $F_{\text{c}}$, activation probability $P_{*,\text{c}}$, conditional mean activation time $\tau_{*,\text{c}}$, as functions of λ for N=6 (blue-solid), 9 (orange-dashed) and 12 (green-dotted) in the KPR model (4.3). We set $f=10^{-2}$, $k_{0,1}=1$, p=0.1, $\mu_{0}=1/240$. While $F_{\text{c}}$ and $P_{*,\text{c}}$ are decreasing functions of λ, $\tau_{*,\text{c}}$ shows non-monotonic behavior. The largest value of $F_{\text{c}}$ is at λ=0 and, for the given parameters, is $F_{\text{c}}=f/\left[f+{\left(\frac{p}{1+p}\right)}^{N-1}\right]\approx 1$. The values of $F_{\text{c}}$, $P_{*,\text{c}}$, $\tau_{*,\text{c}}$ are computed from (4.17), (4.15b), (4.16), respectively.

**Fig. 8:**
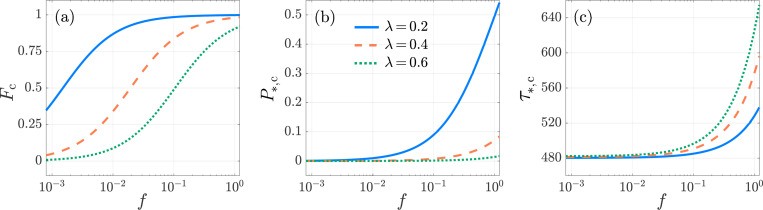
Kinetic proofreading, Neumann boundary conditions. The activation specificity $F_{\text{c}}$, activation probability $P_{*,\text{c}}$, and conditional mean activation time $\tau_{*,\text{c}}$, as functions of f for λ=0.2 (blue-solid), λ=0.4 (orange-dashed) and λ=0.6 (green-dotted) in the KPR model (4.3). We set N=6, $k_{0,1}=1$, p=0.1, $\mu_{0}=1/240$. The values of $F_{\text{c}}$, $P_{*,\text{c}}$, $\tau_{*,\text{c}}$ are computed from (4.17), (4.15b), (4.16), respectively. All curves are monotonically increasing.

**Fig. 9:**
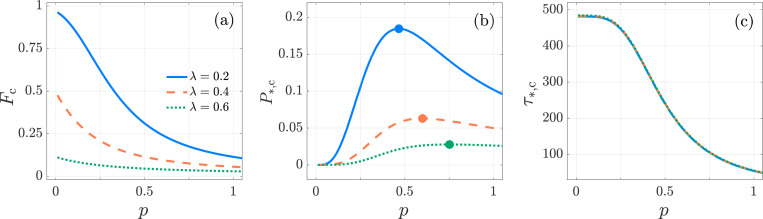
Kinetic proofreading, Neumann boundary conditions. The activation specificity $F_{\text{c}}$, activation probability $P_{*,\text{c}}$, and conditional mean activation time $\tau_{*,\text{c}}$ as functions of p for λ=0.2 (blue-solid), 0.4 (orange-dashed) and 0.6 (green-dotted) in the KPR model (4.3). We set N=6, $f=10^{-2}$, $k_{0,1}=1$, $\mu_{0}=1/240$. In panel (a), $F_{\text{c}}$ decreases with p; its largest value is at p=0 and is $F_{\text{c}}=f/\left[f+\lambda^{N-1}\right]$. The maximum of $P_{*,\text{c}}$ in panel (b) is at $p=p_{\max}$ (4.18) and is represented by filled circles. In panel (c), $\tau_{*,\text{c}}$ exhibits similar trends as a function of p for all three λ values, leading to nearly indistinguishable curves. The values of $F_{\text{c}}$, $P_{*,\text{c}}$, $\tau_{*,\text{c}}$ are computed from (4.17), (4.15b), (4.16), respectively.

**Fig. 10:**
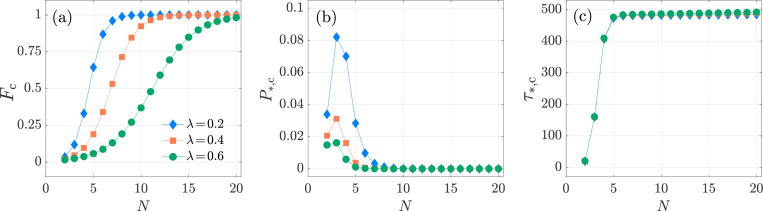
Kinetic proofreading, Neumann boundary condition. The activation specificity $F_{\text{c}}$, activation probability $P_{*,\text{c}}$, and conditional mean activation time $\tau_{*,\text{c}}$ as functions of N≥2 for λ=0.2 (blue-diamond markers), 0.4 (orange-square markers) and 0.6 (green-circular markers) in the KPR model (4.3). We set $f=10^{-2}$, $k_{0,1}=1$, p=0.1, $\mu_{0}=1/240$. In panel (a), $F_{\text{c}}$ increases with N and approaches its limiting value of one as N→∞. In panel (b), $P_{*,\text{c}}$ attains its maximum at $N_{\max}=3$ as per (4.19) for all three values of λ. In panel (c), $\tau_{*,\text{c}}$ exhibits similar trends as a function of p for all three λ values, leading to nearly indistinguishable curves. The values of $F_{\text{c}}$, $P_{*,\text{c}}$, $\tau_{*,\text{c}}$ are computed from (4.17), (4.15b), (4.16), respectively.

As expected, $F_{\text{c}}$ does not depend on the degradation rate $\mu_{0}$. After the initial binding, in the limit λ→1, the dynamics along the two APC chains are equivalent and $F_{\text{c}}\to f/(f+1)\ll 1$ is the ratio of the initial binding rate of the T cell on the cAPC arm, compared to the total initial binding rate on either APC. $F_{\text{c}}$ is a decreasing function of λ, which can be expected since decreasing λ hinders the ability of T cells to detach from the cAPC chain, increasing the likelihood of activation by the cAPC. Interestingly, for fixed λ<1, $F_{\text{c}}$ is a decreasing function of p; lower values of p increase the time that a T cell is bound to both APC chains, however, since λ<1, detachment is more likely along the nAPC chain than along the cAPC chain, resulting in a higher likelihood of activation by the cAPC. Note that under the assumption λ>1, the opposite would hold, and $F_{\text{c}}$ would be an increasing function of p. Finally, as expected $F_{\text{c}}$ is an increasing function of f and an increasing function of N (if λ<1). These trends are shown in Figs. 7 to 10. To achieve high specificity, *i.e.* to obtain $F_{\text{c}}\to 1$, one must utilize small values of λ, p and/or high values of f, N.

Although $\mu_{0}$ does not affect $F_{\text{c}}$, it does reduce $P_{*,\text{c}}$, as shown in (4.15b). Upon differentiating the latter with respect to p (or N) and keeping all other quantities fixed, we find that $P_{*,\text{c}}$ displays maxima at $p=p_{\max}$ and $N=N_{\max}$ as follows

(4.18) $$p_{\max}=\frac{1}{{\left[\frac{k_{0,1}}{\mu_{0}}\left(\frac{1}{\lambda }-1\right)\right]}^{1/N}-1},\;\text{if}\;\lambda <\frac{k_{0,1}}{k_{0,1}+\mu_{0}},$$

(4.19) $$N_{\max}=1+\left\lfloor \frac{\ln\left(\frac{k_{0,1}}{\mu_{0}}\cdot \frac{\ln(1+p)-\ln(\lambda +p)}{\ln(\lambda +p)-\ln p}\right)}{\ln(1+p)-\ln p}\right\rfloor ,\;\;\text{if}\;\lambda <-p+p^{\frac{\mu_{0}}{k_{0,1}+\mu_{0}}}{\left(1+p\right)}^{\frac{k_{0,1}}{k_{0,1}+\mu_{0}}}.$$

Here, $\left\lfloor x\right\rfloor$ represents the floor function of x. Similarly, it can be verified that $P_{*,\text{c}}$ decreases with $\lambda ,\mu_{0}/k_{0,1}$ and increases with f. The activation probability $P_{*,\text{c}}$ is plotted as a function of λ, f, p, N in Figs. 7 to 10. Fig. 7 reveals that the conditional mean activation time $\tau_{*,\text{c}}$ has a maximum in λ. While it is not feasible to determine analytical expressions for the value of λ that maximizes $\tau_{*,\text{c}}$ from (4.16), we note that for small λ, increasing λ is equivalent to a higher likelihood of T cells detaching from the cAPC chain, extending the time required for activation. However, as λ continues to increase, competition from the nAPC chain grows and T cells must bind to the cAPC more rapidly, leading to decreasing values of $\tau_{*,\text{c}}$. Finally, Figs.8 to 10 show that $\tau_{*,\text{c}}$ is a monotonic function of f, N (increasing) and of p (decreasing). We can also compare $\tau_{*,\text{c}}$ and $\tau_{*,\text{n}}$ using (4.16) to write

(4.20) $$\tau_{*,\text{c}}-\tau_{*,\text{n}}=\frac{\left(1-\lambda )(N-1\right)}{\left(\lambda +p)(1+p\right)}.$$

For λ<1, $\tau_{*,\text{c}}$ is larger than $\tau_{*,\text{n}}$ and vice-versa for λ<1. Interestingly, $\tau_{*,\text{c}}=\tau_{*,\text{n}}$ for λ=1 regardless of the value of f. Although the likelihood that a T cell initially binds to a cAPC is lower than that of initially binding to a nAPC, once it has bound, if the return rates along both arms are equal (λ=1), then, on average, the time it takes for a T cell to reach the final state of either the cAPC or nAPC arm, will be the same. The difference in (4.20) varies linearly with N and is appreciable only for large enough N. How $F_{\text{c}}$, $P_{*,\text{n}}$, $P_{*,\text{c}}$ depend on relevant parameters while the others are kept fixed is summarized in Table 1. The corresponding trends for $\tau_{*,\text{n}}$ and $\tau_{*,\text{c}}$ will depend on specific parameter choices when these quantities are considered as functions of λ, $k_{0,1}$ or f; both $\tau_{*,\text{n}}$ and $\tau_{*,\text{c}}$ will instead decrease with $\mu_{0}$, p and increase with N, regardless of other parameters.

### 4.2. Robin (partially reflecting) boundary conditions.

We now analyze the KPR model under partially reflecting boundary conditions, following the same approach used in section 3.4. Specifically, we transform the PDE system (4.3) into an IDE for $\rho_{0}(x,t)$ which, we will show, has the same form as (3.33) and where the details of the KPR-dynamics are embedded in a new kernel $K_{\text{KP}}(t)$. We begin by noting that contrary to the kinetic matrices $M_{\text{c}}$ and $M_{\text{n}}$ used in section 3.4, the kinetic matrices $B_{\text{c}}$ and $B_{\text{n}}$ in (4.4) are not diagonalizable. We thus apply the Laplace transform to each equation in (4.3b) and (4.3c) to obtain

(4.21a) $$n_{i}(x,s)=\frac{k_{0,1}p^{i-1}}{{\left(s+1+p\right)}^{i}}\rho_{0}(x,s),\;\text{for}\;1\leq i\leq N-1,$$

(4.21b) $$c_{i}(x,s)=\frac{fk_{0,1}p^{i-1}}{{\left(s+\lambda +p\right)}^{i}}\rho_{0}(x,s),\;\text{for}\;1\leq i\leq N-1.$$

Upon adding all expressions for $n_{i}(x,s)$ in (4.21a) and $c_{i}(x,s)$ in (4.21b) we find

(4.22) $$\begin{array}{l} \sum_{i=1}^{N-1}n_{i}(x,s)=\frac{k_{0,1}}{s+1}\left[1-{\left(\frac{p}{s+1+p}\right)}^{N-1}\right]\rho_{0}(x,s),\\ \sum_{i=1}^{N-1}c_{i}(x,s)=\frac{fk_{0,1}}{s+\lambda }\left[1-{\left(\frac{p}{s+\lambda +p}\right)}^{N-1}\right]\rho_{0}(x,s). \end{array}$$

The inverse Laplace transform (4.22) can be written as

(4.23) $$\begin{array}{l} \sum_{i=1}^{N-1}n_{i}(x,t)=\int_{0}^{t}K_{\text{n}}\left(t-t^{\prime }\right)\rho_{0}\left(x,t^{\prime }\right)\text{d}t{,}^{\prime }\\ \sum_{i=1}^{N-1}c_{i}(x,t)=\int_{0}^{t}K_{\text{c}}\left(t-t^{\prime }\right)\rho_{0}\left(x,t^{\prime }\right)\text{d}t^{\prime }, \end{array}$$

where the kernels $K_{\text{n}}(t)$ and $K_{\text{c}}(t)$ are the inverse Laplace transforms of

(4.24) $$K_{\text{n}}(s)=\frac{k_{0,1}}{s+1}\left[1-{\left(\frac{p}{s+1+p}\right)}^{N-1}\right],\;K_{\text{c}}(s)=\frac{\lambda fk_{0,1}}{s+\lambda }\left[1-{\left(\frac{p}{s+\lambda +p}\right)}^{N-1}\right],$$

respectively. Eqs. 4.24 define the KPR memory kernel $K_{\text{KPR}}(t)$

(4.25) $$K_{\text{KPR}}(t)=K_{\text{n}}(t)+K_{\text{c}}(t).$$

Finally, by substituting (4.23) into (4.3), we re-obtain the IDE (3.33) with the KPR memory kernel (4.25), $K(t)\to K_{\text{KPR}}(t)$. Using the methods described in section B we derive the corresponding $\rho_{0}(x,t)$, and obtain $c_{N-1}(x,t)$ using (4.21b). Expressions (4.6)–(4.10) allow us to compute $P_{*,\text{c}}$ and $\tau_{*,\text{c}}$. Notably, the resulting $F_{\text{c}}$ is independent of κ and still given by (4.17). To show this, we integrate (4.21a) and (4.21b) spatially over Ω for i=N−1, and evaluate the resulting expressions at s=0 to find

(4.26) $${\bar{n}}_{N-1}(s=0)=\frac{k_{0,1}p^{N-2}{\bar{\rho }}_{0}(s=0)}{{\left(1+p\right)}^{N-1}},\;{\bar{c}}_{N-1}(s=0)=\frac{fk_{0,1}p^{N-2}{\bar{\rho }}_{0}(s=0)}{{\left(\lambda +p\right)}^{N-1}}.$$

Substituting these expressions into (4.7) leads to

(4.27) $$P_{*,\text{n}}=k_{0,1}{\bar{\rho }}_{0}(s=0){\left(\frac{p}{1+p}\right)}^{N-1},\;P_{*,\text{c}}=fk_{0,1}{\bar{\rho }}_{0}(s=0){\left(\frac{p}{\lambda +p}\right)}^{N-1}.$$

**Fig. 11:**
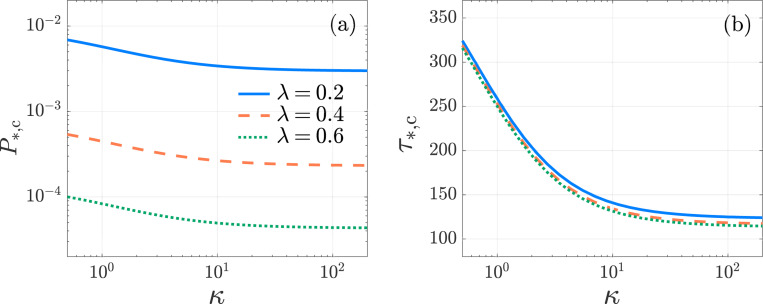
Kinetic proofreading, Robin boundary conditions. The activation probability $P_{*,\text{c}}$ and the conditional mean activation time $\tau_{*,\text{c}}$ as functions of the Robin coefficient κ in the KPR model (4.3) with λ=0.2 (blue-solid), 0.4 (orange-dashed), and 0.6 (green-dotted). We set N=6, $\mu_{0}=1/240$, $f=10^{-2}$, $k_{0,1}=1$, $D=1.8\times 10^{-3}$. The values of $P_{*,\text{c}}$, $\tau_{*,\text{c}}$ are computed from (4.15b), (4.16), respectively. The activation specificity $F_{\text{c}}$ in (4.17) is independent of κ. All curves are monotonically decreasing.

From the definition of $F_{\text{c}}$ given in (4.11) it follows that (4.17) still holds and that $F_{\text{c}}$ is independent of ${\bar{\rho }}_{0}(s=0)$ and of κ. Similar to the results for the multi-stage two-arm model in section 3, $P_{*,\text{n}}$, $P_{*,\text{c}}$, and $\tau_{*,\text{c}}$ decrease as the Robin coefficient κ increases. Larger values of κ imply a higher likelihood that the T cell exits the T cell zone, resulting in decreases in both $P_{*,\text{n}}$ and $P_{*,\text{c}}$. Furthermore, larger values of κ require a T cell to reach its cAPC in a shorter time, decreasing $\tau_{*,\text{c}}$. These trends are confirmed in Fig. 11 where $P_{*,\text{c}}$ and $\tau_{*,\text{c}}$ are observed to both decrease with κ, for various values of λ. Although $P_{*,\text{c}}$ varies by about an order of magnitude across the different choices of λ in Fig.11, the corresponding $\tau_{*,\text{c}}$ are of similar scale.

## 5. Discussion and Conclusions.

We constructed and analyzed a diffusion-reaction model to describe T cells diffusing while seeking for their cognate APC target among a sea of noncognate APCs. The search process is delayed by interactions with nAPCs, and hindered by T cell death and escape from the T cell zone compartment. Our results show that when T cells and APCs bind through a sequence of N steps, the activation probability $P_{*}$ increases with the forward-to-backward ratio p/q. The bias toward forward transitions along the APC chains decreases the likelihood of degradation while in the free state, and increases the likelihood that the final, activation state N is reached.

How various parameters affect the conditional mean activation time $\tau_{*}$ is more subtle. Within certain parameter regimes, increasing p may prolong interactions between T cells and nAPCs, thereby increasing $\tau_{*}$. While $P_{*}$ consistently decreases with the number of states N, $\tau_{*}$ always increases with N. As can be expected, a larger N reduces the likelihood of activation but extends the time required for it to occur. Diffusion does not directly promote activation but facilitates transport towards the boundary, increasing the likelihood of escape if the boundary is not fully reflecting. As a result, both $P_{*}$ and $\tau_{*}$ decrease with increasing D.

We also considered a variant T cell activation scheme in which reaching the end of either the cAPC or nAPC interaction chain can activate the T cell, but only in the first case in a successful manner. In this scheme, at each intermediate state along both the nAPC and cAPC arms the T cell can reset back to the free state. This kinetic proofreading scheme biases the system toward successful cAPC activation, even when the cAPC resetting rate is only modestly lower than that of the nAPCs.

Mathematically, we studied an integro-differential equation with a memory kernel derived from the multi-stage or KPR kinetics. This IDE reduces to an ODE under Neumann boundary conditions. More general kernels could be incorporated, using simplified or alternative kinetics that account for differential lengths of the cAPC and nAPC arms, heterogeneous forward and backward rates, and distinct nAPCs. We also assumed that APC are uniformly distributed within the T cell zone; this assumption could be modified to allow for heterogeneity in the spatial distribution of APCs within the T cell zone. Couplings between lymph nodes and the vascular network could also be included, to study T cells circulating through the lymphatic system.

## Figures and Tables

**Table 1:** Trends of $F_{\text{c}}$, $P_{*,\text{c}}$, $P_{*,\text{n}}$ $\tau_{*,\text{c}}$ and $\tau_{*,\text{n}}$ as functions of λ, p, $\mu_{0}$, $k_{0,1}$, f, and N. Note that $F_{\text{c}}$ is independent of $\mu_{0}$, $k_{0,1}$ and that $P_{*,\text{c}}$ and $P_{*,\text{n}}$ depend only on the ratio $\mu_{0}/k_{0,1}$. Universal trends for $\tau_{*,\text{c}}$ and $\tau_{*,\text{n}}$ can only be determined for p, $\mu_{0}$, N. Both quantities are decreasing functions of p, $\mu_{0}$ and increasing functions of N if all other parameters are kept fixed. Whether $\tau_{*,\text{c}}$ and $\tau_{*,\text{n}}$ increase or decrease as functions of λ, $k_{0,1}$, f depends on parameter choices.

	λ	p	$\mu_{0}$	$k_{0,1}$	f	N
$F_{\text{c}}$ (4.17)	decrease	decrease	−	−	increase	increase
$P_{*,\text{c}}$ (4.15b)	decrease	max at $p=p_{max}$	decrease	increase	increase	max at $N=N_{max}$
$P_{*,\text{n}}$ (4.15a)	increase	increase	decrease	increase	decrease	decrease
$\tau_{*,\text{c}}$ (4.16)		decrease	decrease			increase
$\tau_{*,\text{n}}$ (4.16)		decrease	decrease			increase

## Data Availability

No experimental data were used in this study. Codes are openly available at https://github.com/kawahtony/t_cell_activation.

## Appendix A. Parameter estimation and physical considerations.

The size of a lymph node gland depends on where in the body it is located; typically it is oval shaped. Under healthy conditions, the long axis ranges between 0.2 to 2.5 cm with an estimated average of 1.5 cm, whereas the typical short axis extends up to 1 cm [13,24,34,43,49,55]. The size of the T cell zone, where most of the interactions between T cells and APCs occur, depends on the lymph node anatomy, and whether it is activated or not, but in general the it occupies a significant portion of the interior of a lymph node. In this paper, we use the two terms interchangeably and a spherical domain for the T cell zone. We set its radius to a=0.1 cm. This estimate is taken by assuming a typical short axis length of 0.4 cm (corresponding to a radius of 0.2cm) and by assuming that the radius of the T cell zone is roughly half that of short radius.

Naïve T cells measure between 5–10 *μ*m in diameter, whereas the size of APCs varies: B cells are in the same range as T cells, mature dendritic cells have a diameter of 10–15 *μ*m and for macrophages the range is 20–50 *μ*m. A single lymph node contains between 10^6^ to 10^7^ T cells; the abundance increases upon activation when the size of the T cells can also expand. The number of APCs residing in a lymph node depends on several factors, including the specific type of antigen, the lymph node type, and whether an infection is under way.

To estimate the dimensional parameters that appear in our model we refer to experimental findings from previous literature. Several imaging studies estimated the diffusivity of T cells to be about D=60 *μ*m^2^min^−1^ [12,39]. On average it is estimated that T cells remain in contact with nAPCs for about 3 minutes before dissociating [4, 39] so we set $K_{1,0}=1/3$ min^−1^. The mean residence time in the lymph node for a T cell is between 12 and 24 hours [18,27,40,53]; thus, we set the exit rate from a lymph node $M_{0}=1/720$ min^−1^. Finally, it is estimated that during a 12 hour residence time in a lymph node, a T cell makes about 160 interactions with nAPCs [18]. Hence, the average time separation between each T cell – nAPC interaction is about 4 and a half minutes. Since we assume that the time a T cell spends bound to a nAPC is 3 minutes, the time spent in the free searching state is roughly one and a half minutes.

## Appendix B. Series solution for integro-differential equations.

We solve the IDE (3.33) for a general kernel K(t). Due to spherical symmetry, we can omit the angular variables and write $\rho_{0}(x,t)=\rho_{0}(r,t)$ so that (3.33), the boundary condition (3.9), and the initial condition (3.4) become

(B.1a)
$$\partial_{t}\rho_{0}=\frac{D}{r^{2}}\partial_{r}\left(r^{2}\partial_{r}\rho_{0}\right)-\left[\mu_{0}+(1+f)k_{0,1}\right]\rho_{0}+\int_{0}^{t}K\left(t-t^{\prime }\right)\rho_{0}\left(r,t^{\prime }\right)\text{d}t^{\prime },$$

(B.1b)
$$\partial_{r}\rho_{0}+\kappa \rho_{0}=0\;\text{at}\;r=1,\;\rho_{0}(r,0)=\frac{\delta (r)}{4\pi r^{2}}.$$

To solve (B.1a) we separate variables by setting $\rho_{0}(r,t)=\phi (r)T(t)$ resulting in two decoupled problems

(B.2a)
$$\frac{{\text{d}}^{2}\phi }{\;\text{d}r^{2}}+\frac{2}{r}\frac{\;\text{d}\phi }{\;\text{d}r}=-\frac{\nu^{2}}{D}\phi ,$$

(B.2b)
$$\frac{\text{d}T}{\;\text{d}t}+\left(\mu_{0}+(1+f)k_{0,1}+\nu^{2}\right)T=\int_{0}^{T}K(t-s)T(s)\text{d}s,$$

where ν is arbitrary. The solution to the spatial equation (B.2a) is

(B.3)
$$\phi (r)=\frac{1}{r}\sin\left(\frac{\nu r}{\sqrt{D}}\right),$$

Applying this form to the boundary condition in (B.1b) constrains ν to be any of the roots of the transcendental equation

(B.4)
$$(\kappa -1)\sin\left(\frac{\nu }{\sqrt{D}}\right)+\frac{\nu }{\sqrt{D}}\cos\left(\frac{\nu }{\sqrt{D}}\right)=0,\;\nu >0.$$

Eq. (B.4) can be rewritten as

(B.5)
$$(\kappa -1)\sin\alpha +\alpha \cos\alpha =0,\;\text{where}\;\alpha =:\frac{\nu }{\sqrt{D}},\;\alpha >0.$$

We enumerate the $\alpha_{n}$ roots of (B.5) in increasing order for n≥1. Once a specific root $\alpha_{n}$ is selected, the related time dependent solution can be determined by setting $\nu =\sqrt{D}\alpha_{n}$ in the temporal equation (B.2b). This leads to a series of solutions $\phi_{n}(r)T_{n}(t)$ associated to the specific root $\alpha =\alpha_{n}$. To proceed, and for simplicity, we set N=1 so that $n_{1}$ and $c_{1}$ can be expressed in terms of $\rho_{0}$ through (3.8b) and (3.8c) as

(B.6a)
$$n_{1}(r,t)=\int_{0}^{t}k_{0,1}e^{-\left(t-t^{\prime }\right)}\rho_{0}\left(r,t^{\prime }\right)\text{d}t^{\prime },$$

(B.6b)
$$c_{1}(r,t)=\int_{0}^{t}fk_{0,1}\rho_{0}\left(r,t^{\prime }\right)\text{d}t^{\prime }.$$

By substituting (B.6a) into (3.8a) the memory kernel K(t) can be explicitly written for N=1 as $K(t)\equiv k_{0,1}e^{-t}$. To find $T_{n}(t)$, we now take the Laplace transform L of (B.2b) for N=1 to find

(B.7)
$$L\left[T_{n}\right](s)=\frac{s+1}{\left(s+\mu_{0}+(1+f)k_{0,1}+D\alpha_{n}^{2}\right)(s+1)-k_{0,1}}$$

Where we used the fact that the Laplace transform of a convolution of two functions is the product of the their Laplace transforms. The RHS of (B.7) is a rational function of the Laplace frequency variable s. Evaluating its inverse Laplace transform yields

(B.8)
$$T_{n}(t)=\frac{1}{s_{n}^{+}-s_{n}^{-}}\left[\left(1+s_{n}^{+}\right)e^{s_{n}^{+}t}-\left(1+s_{n}^{-}\right)e^{s_{n}^{-}t}\right]$$

where $s_{n}^{\pm }$ are the (negative) roots of

(B.9)
$$s^{2}+\left(\mu_{0}+(1+f)k_{0,1}+D\alpha_{n}^{2}+1\right)s+\left(\mu_{0}+fk_{0,1}+D\alpha_{n}^{2}\right)=0.$$

Upon rearranging terms we can write

(B.10)
$$T_{n}(t)=e^{-h_{n}t}\left[\frac{1-h_{n}}{H_{n}}\sinh\left(H_{n}t\right)+\cosh\left(H_{n}t\right)\right]$$

where $h_{n}=\frac{1}{2}\left(\mu_{0}+(1+f)k_{0,1}+D\alpha_{n}^{2}+1\right)$ and $H_{n}=\frac{1}{2}{\left[\mu_{0}+(1+f)k_{0,1}+D\alpha_{n}^{2}+1\right)}^{2}{\left.-4\left(\mu_{0}+fk_{0,1}+D\alpha_{n}^{2}\right)\right]}^{1/2}$. The solution in (B.10) satisfies $T_{n}(t=0)=1$. The overall solution can thus be written as a linear combination of products $\phi_{n}(r)T_{n}(t)$ where the amplitudes $A_{n}$ are determined by the spatial initial condition. We thus have

(B.11)
$$\rho_{0}(r,t)=\sum_{n=1}^{\infty }A_{n}\phi_{n}(r)T_{n}(t),\;\text{where}\;\sum_{n=1}^{\infty }A_{n}\phi_{n}(r)=\frac{\delta (r)}{4\pi r^{2}}.$$

The $\phi_{n}(r)$ functions represent an orthogonal basis for r∈[0,1] since, given n≠m

(B.12a)
$$\int_{0}^{1}\phi_{n}(r)\phi_{m}(r)4\pi r^{2}\;\text{d}r=4\pi \int_{0}^{1}\sin\left(\alpha_{n}r\right)\sin\left(\alpha_{m}r\right)\text{d}r,$$

(B.12b)
$$=4\pi \frac{\alpha_{m}\cos\alpha_{m}\sin\alpha_{n}-\alpha_{n}\cos\alpha_{n}\sin\alpha_{m}}{\alpha_{n}^{2}-\alpha_{m}^{2}}=0$$

where the last equality in (B.12b) follows from both $\alpha_{n}$, $\alpha_{m}$ satisfying the boundary condition in (B.5). For n≠0

(B.13)
$$g_{n}^{2}=:\int_{0}^{1}\phi_{n}^{2}(r)4\pi r^{2}\;\text{d}r=4\pi \int_{0}^{1}\sin{\left(\alpha_{n}r\right)}^{2}\;\text{d}r=\frac{\pi \left[2\alpha_{n}-\sin\left(2\alpha_{n}\right)\right]}{\alpha_{n}}.$$

Since $\lim_{r\to 0}\phi_{n}(r)=\alpha_{n}$ we can write

(B.14)
$$A_{n}=\frac{\alpha_{n}}{g_{n}^{2}}=\frac{1}{\pi }\frac{\alpha_{n}^{2}}{\left[2\alpha_{n}-\sin\left(2\alpha_{n}\right)\right]}\;\text{for}\;n\neq 0$$

so that

(B.15)
$$\rho_{0}(r,t)=\frac{1}{\pi }\sum_{n=1}^{\infty }\frac{\alpha_{n}^{2}\sin\left(\alpha_{n}r\right)e^{-h_{n}t}}{\left(2\alpha_{n}-\sin\left(2\alpha_{n}\right)\right)r}\left[\frac{1-h_{n}}{H_{n}}\sinh\left(H_{n}t\right)+\cosh\left(H_{n}t\right)\right].$$

It is straightforward to verify that $\lim_{t\to \infty }\rho_{0}(r,t)=0$ for all r values. The expression in (B.15) can be inserted into Eqs. (B.6a), (B.6b) to find $n_{1}(r,t)$ and $c_{1}(r,t)$ respectively, leading to the asymptotic limits $\lim_{t\to \infty }\rho_{0}(r,t)=\lim_{t\to \infty }n_{1}(r,t)=0$ and

(B.16)
$$\underset{t\to \infty }{\lim}c_{1}(r,t)=\frac{1}{\pi }\sum_{n=1}^{\infty }\frac{\alpha_{n}^{2}\sin\left(\alpha_{n}r\right)}{\left[2\alpha_{n}-\sin\left(2\alpha_{n}\right)\right]r}\left[\frac{fk_{0,1}}{\mu_{0}+fk_{0,1}+D\alpha_{n}^{2}}\right]$$

We can calculate the activation probability $P_{*}$ under the Robin boundary condition by evaluating the spatial integral in (B.16)

(B.17)
$$P_{*}=\underset{t\to \infty }{\lim}\int_{0}^{1}c_{1}(r,t)4\pi r^{2}\;\text{d}r.$$

Upon inserting (B.16) into (B.17) and evaluating the integral we find

(B.18)
$$P_{*}=4\kappa \sum_{n=1}^{\infty }\frac{\sin\alpha_{n}}{2\alpha_{n}-\sin\left(2\alpha_{n}\right)}\left[\frac{fk_{0,1}}{\mu_{0}+fk_{0,1}+D\alpha_{n}^{2}}\right].$$

As κ→0 one can show that $\alpha_{1}\to 0$ as well and that $\lim_{\kappa \to 0}\kappa \sin\left(\alpha_{1}\right)/\left(2\alpha_{1}-\right.\left.\sin\left(2\alpha_{1}\right)\right)=1/4$. All other terms in (B.18) converge to zero as κ→0 so that (B.18) reduces to

(B.19)
$$\underset{\kappa \to 0}{\lim}P_{*}=\underset{t\to \infty }{\lim}\int_{0}^{1}c_{1}(r,t)4\pi r^{2}\;\text{d}r=\frac{fk_{0,1}}{\mu_{0}+fk_{0,1}}.$$

Thus, under perfectly reflecting, Neumann boundary conditions, the single-stage $\left(N=1\right)$ activation probability is simply the ratio of the binding rate between a T cell and its cAPC, to the total rate at which the T cell reaches any of its absorbing states, either degradation or binding to its cAPC. The methods presented here are also applicable to the general multi-stage case with N>1, where the memory kernel K(t) is given by (3.34), and to the KPR model, where the memory kernel $K_{\text{KPR}}(t)$ is given by (4.25).

## Appendix C. Mean time of engagement between T cells and nAPCs.

Here, we estimate the interaction time between a T cell and a nAPC by determining the mean first time $\tau_{\text{nAPC}}$ for a T cell at the first stage of engagement ${\text{N}}_{1}$ to return to the free state ${\text{T}}_{0}$. For concreteness, we focus on the nAPC arm, as extracted from

(C.1)
$${\text{T}}_{0}\;\underset{K_{1,0}}{\overset{K_{\text{n}}}{⇌}}\;{\text{N}}_{1}\;\underset{Q}{\overset{P}{⇌}}\;{\text{N}}_{2}\;\underset{Q}{\overset{P}{⇌}}\;\cdots \;\underset{Q}{\overset{P}{⇌}}\;{\text{N}}_{N-1}\;\underset{Q}{\overset{P}{⇌}}\;{\text{N}}_{N}$$

To determine $\tau_{\text{nAPC}}$ we first write the equations for the probability $P={\left(P_{1},\ldots ,P_{N}\right)}^{T}$ for a T cell and a nAPC to be bound at state (1,…,N), respectively, and the probability $P_{0}$ for the T cell to remain free, respectively. Since we are not interested in transport phenomena [21], the dynamics follow (3.2b) without the spatial components. The forward equation is written as

(C.2)
$$\partial_{t}P_{0}=P_{1}-\left(\mu_{0}+k_{0,1}\right)P_{0},\;\partial_{t}P=M_{\text{n}}P+k_{0,1}P_{0}e_{1}.$$

We assume that at t=0 the T cell is at state n=1 so that $P_{1}(t=0)=1$ and $P_{0}(t=0)=P_{i\neq 1}(t=0)=0$. We also define the survival probability $S={\left(S_{1},\ldots ,S_{N}\right)}^{T}$ as the likelihood that having started at state (1,…,N) at t=0 the T cell is still engaged to any nAPC state at time t. Thus, the likelihood of the free T cell being bound to the nAPC at any time t is $S_{0}(t)=0$. Furthermore if we assume the T cell is initially at its first binding state then $S_{1}(t=0)=1$ and $S_{i\neq 1}(t=0)=0$. It is well known that the survival probability follows the backward equation stemming from $M_{\text{n}}^{†}$, the adjoint of $M_{\text{n}}$ so that

(C.3)
$$\partial_{t}S_{0}=0,\;\partial_{t}S=M_{\text{n}}^{†}S.$$

The survival time distribution is given by $-\partial_{t}S$. As a result, the mean first passage time $T={\left(T_{1},\ldots ,T_{N}\right)}^{T}$ of a partially bound T cell starting from stage (1,…,N) to the free state can derived as

(C.4)
$$T=-\int_{0}^{\infty }t\partial_{t}S\;\text{d}t=\int_{0}^{\infty }S\text{d}t.$$

Upon integrating the right-hand expression in (C.3) with respect to time, from t=0 to t→∞, and by imposing S(t=0)=1 and S(t→∞)=0 we find $-I=M_{\text{n}}^{†}T$, where I is the identity matrix. The inverse $T=-{\left(M_{\text{n}}^{†}\right)}^{-1}I$ defines the mean first passage time $T_{1}\equiv \tau_{\text{nAPC}}$ for the T cell to be in the free, unbound state starting from the first bound stage. Using standard matrix inversion methods we find

(C.5)
$$\tau_{\text{nAPC}}=T_{1}=\frac{1-{\left(p/q\right)}^{N}}{1-p/q}.$$

Note that $\lim_{p/q\to 1}T_{1}=N$. As evaluated in (C.5), $T_{1}$ is an increasing function of p/q, so that the higher the bias away from the free state and towards higher binding stages, the larger $T_{1}$.

## Appendix D. Spectral analysis of the kinetic matrices.

Here, we show that the kinetic matrices $M_{\text{n}}$ and $M_{\text{c}}$ are diagonalizable and that they can be expressed according to the eigendecomposition in (3.31). One of the conditions for any n×n matrix to be diagonalizable is that it must have n distinct eigenvalues. For convenience, we denote the (N−1)×(N−1) submatrix of $M_{\text{c}}$ as ${\tilde{M}}_{\text{c}}$ and denote its characteristic polynomial as ${\tilde{p}}_{\text{c}}(\lambda )$. When p, q>0, $M_{\text{n}}$ and ${\tilde{M}}_{\text{c}}$ are non-singular Jacobi matrices, whose characteristic polynomials have distinct, real and non-zero roots (see Chapter 2 of Ref. [16]). Particularly, $M_{\text{n}}$ is diagonalizable. A direct calculation shows that the characteristic polynomial of $M_{\text{c}}$, $p_{\text{c}}(\lambda )=\lambda {\tilde{p}}_{\text{c}}(\lambda )$, has distinct, real and non-zero roots, implying that it also has distinct, real eigenvalues, one of them being zero. The matrix $M_{\text{c}}$ is therefore diagonalizable. In conclusion, the kinetic matrices $M_{\text{n}}$ and $M_{\text{c}}$ are diagonalizable provided that the forward and backward binding rates p, q are positive.

## Appendix E. Further reading.

Background on T cell activation can be found in [5, 18, 26, 30, 45]. Experimental imaging studies of T cell–APC interactions are reported in [37–39,50]. Mathematical and computational models of T cell migration are presented in [12, 20, 46]. First-passage time theory is introduced in [9, 25, 48]. Kinetic proofreading was proposed in [22,42] and applied to antigen recognition in [36].
